# Effective Tuning of the Performance of Conductive Silicon Compound by Few-Layered Graphene Additives

**DOI:** 10.3390/nano12060907

**Published:** 2022-03-09

**Authors:** Zhensheng Wu, Haitao Yang, Fuqiang Tian, Hao Ren, Yu Chen

**Affiliations:** 1 School of Electrical Engineering, Beijing Jiaotong University, Beijing 100044, China; zhshwu@bjtu.edu.cn (Z.W.); fqtian@bjtu.edu.cn (F.T.); 2 School of Materials Science and Engineering, China University of Petroleum (East China), Qingdao 266580, China; renh@upc.edu.cn; 3 Hegang Zhenjin Graphene New Material Institute, Hegang 154100, China; cy374345509@163.com

**Keywords:** electrical joint compound, mechanically exfoliated graphene, volumetric resistivity, frictional properties

## Abstract

Electric conductive silicon compounds are widely used and essential in electric power, energy and information industries. However, there are still problems such as insufficient stability of physical and chemical properties and weak electrical conductivity. To address the problem of low contact reliability of electrical joints in high-power transmission and distribution equipment, we assessed the influence of mechanically exfoliated graphene (MEG) content on the physicochemical properties of electrical joint compound (EJC). Varying amounts of few-layer MEG prepared with the conventional mechanically exfoliated method was added to the conductive silicon compounds, of which various physicochemical properties, such as penetration, drip point, volume resistivity and frictional properties were systematically assessed and compared with those with copper additive. We found that the addition of MEG effectively enhanced the temperature and mechanical stability of EJC and significantly reduced the material volume resistivity. This work paves the way to improve the key performance of electric conductive silicon compounds with advanced nanomaterials.

## 1. Introduction

With a growth of modern ultrahigh voltage transmission coverage, the load of high-voltage transmission and substation equipment also grows rapidly. After long-term operation, the equipment usually contracts the problems of contact reliability decreasing and overheating [1,2,3]. In recent years, electrical joint compounds (EJCs) have been proposed as a promising alternative to enhance the electrical contact reliability. EJCs are mainly composed of base oil, conductive filler and thickener, which are mainly used to increase the actual effective contact area by coating the contact surface of the equipment, thus reducing the electrical contact resistance, hindering the energy consumption and overheating of electrical joints [4]. In addition, the power compound also acts a sealing effect on the electrical contact surface which prevents water vapor, corrosive gases and dust from penetrating into the electrical contact surfaces, causing corrosion, aging and wear, and improves the lifetime of the electrical connections [5,6,7].

In addition to high-temperature stability and moderate hardness, high-quality EJC should also have good electrical conductivity and appropriate friction properties, and the latter two properties are more difficult to improve. In recent years, the frictional and conductivity enhancement of EJC has attracted tremendous research interests. The physical and chemical properties of the EJC can be effectively optimized by improving the additives in the EJC.

Some researchers have improved the conductive fillers by choosing nanometal materials to increase the specific surface area of the fillers and thus enhance their contact properties. Ge, X.Y. et al. synthesized a new conductive grease with Sb-doped SnO_2_ (ATO) as an additive, and ATO can significantly improve the tribological properties of the grease [8]. Kang, H. et al. investigated the composite grease of copper nanopowder and silicone oil and found that the addition of copper nanopowder greatly enhanced the thermal conductivity [9].

Carbon-based materials have not only high electrical conductivity but also larger specific surface area and lower cost compared to nanometal particles, so it becomes an important means to improve the performance of EJC. Cao, Z.F. et al. used Ketjen black (KB), acetylene black (AB) and carbon black (CB) to prepare conductive grease and found that the conductive grease prepared with KB had better frictional properties and excellent electrical conductivity under various temperature environments [10]. Liu, H.T. et al. and Ge, X.Y. et al. investigated the effects of carbon nanotubes as conductive additives on the electrical conductivity and tribological properties of greases; the results showed that carbon nanotubes could greatly reduce the bulk resistivity of the base grease while improving the tribological properties of the base grease to some extent [11,12]. Qu, Y.H. et al. and Lim, S. et al. investigated the electrical conductivity of graphene and the effect of copper paste incorporation on the microstructure and properties of EJC; the results showed that graphene incorporation resulted in lower rheological properties and higher electrical conductivity of EJC [13,14]. Liang, W. et al. used reduced graphene oxide (RGO) as a substrate to improve thermal grease performance and enhance thermal management by self-assembly of hBN nanosheets on the RGO surface [15]. From the above case, the addition of carbon-based materials such as graphene can favorably affect the various properties of EJC.

Mechanically exfoliated few-layered graphene has low defect density, low cost and, more importantly, its preparation process is environmentally friendly. Combined with its chemical stability and high thermal and electric conductivity, MEG would be a promising additive to improve the key performance of silicon EJCs. In this paper, we use MEG as an additive to tune the physicochemical properties of EJCs. Various measurements, such as penetration, drip point, volume resistivity and frictional properties, were conducted to systematically characterize the performance of the resulted EJCs; we found that the addition of MEG is highly effective in improving drip point and frictional properties, as well as reducing penetration and volume resistivity.

## 2. Materials and Methods

### 2.1. Materials

Main materials for MEG preparation: expanded graphite (C, 0.05 mm) produced by Risheng Graphite Co., Ltd. (Qingdao, China); sodium carboxymethyl cellulose (CMC) for graphene slurry dispersion; filtered pure water prepared by laboratory water purifier (conductivity 4.26 μS/cm).

Main materials for EJC: phenylmethyl silicone oil 255#-300CS (viscosity 300 mm^2^/s 25 °C, relative density 1.020–1.080, flashpoint ≥300 °C, freezing point ≤−40) produced by Xinda Chemical Co., Ltd. (Bengbu, China); irregular copper powder (electrolytic, 2 μm) produced by Zhuyu Alloy Material Co., Ltd. (Nangong, China); graphite powder (1000 mesh, particle size 13 μm) produced by Hegang Zhenjin Graphene New Material Research Institute (Hegang, China).

### 2.2. Preparation of Graphene

**Figure 1:**
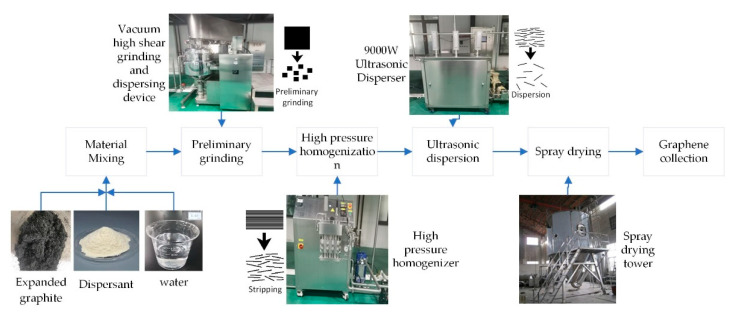
Preparation process of mechanically exfoliated graphene.

Few-layered graphene is prepared by the mechanically exfoliated method, with the main consideration to break the van der Waals interactions between the graphite layers by mechanical shear, impact and cavitation. The MEG preparation process is shown in Figure 1.

(1) Preliminary grinding: After weighing 2 kg of expanded graphite, 47.5 kg of pure water and 0.5 kg of CMC, the raw material was poured into the vacuum high shear grinding and dispersing device, with 2200 r/min for 2 h. During the time, the grinding tank made planetary stirring to the expanded graphite, which generates strong mixing shear force through high-speed tumbling and friction of raw materials to achieve the purpose of prestripping.

(2) High-pressure homogenization: The initially sheared ground graphene feedstock was moved into the high-pressure homogenizer through a preset pipeline. The pressure was set at 80 MPa for 1 h. The purpose of this operation is to further disrupt the van der Waals forces between graphite layers and form few-layer graphene slurry. This step was repeated 5-10 times until the graphene was exfoliated to about 5 layers.

(3) Ultrasonic dispersion: The graphene slurry was pressed into the ultrasonic device by air pressure and dispersed in a 9000 watts ultrasonic device. The ultrasonic device was designed to further disperse the flake graphene and prevent the appearance of graphene agglomerates.

(4) Spray drying: The completely separated graphene slurry was transferred to the spray drying tower, and spray drying was carried out at 90 °C. After this, the graphene slurry was transformed into dry solid graphene powder, and finally the few-layer graphene powder was obtained in the drying tower collection device.

### 2.3. Preparation of EJC

(1) Formulation determination: To investigate the effect of adding mechanically exfoliated MEG on the physicochemical properties of kinetic composite grease, two sets of formulations were prepared, pure copper powder and copper powder + MEG, in which copper powder was the base filler and MEG was the additional filler. Meanwhile, considering the large difference in the volume to mass ratio between MEG and copper powder, the copper powder + graphite formulation was added as a control group to further improve the experimental persuasiveness. Considering the limitation of the softness and hardness of the slurry, the filler ratio was limited to 77–81 wt% according to engineering experience, and the formulation was adjusted by 1 wt% of the filler content; different filler contents are indicated by using I, II, III, IV and V. The final obtained three groups of conductive grease conductive filler in proportion to the total mass are as follows: copper powder type, copper powder accounted for 77, 78, 79, 90 and 81 wt%; copper powder + graphite type, copper powder accounted for 76 wt%, graphite accounted for 1, 2, 3, 4, and 5 wt%; copper powder + MEG type, copper powder accounted for 76 wt%, MEG accounted for 1, 2, 3, 4, and 5 wt%.

(2) Material pretreatment: We weighed a certain amount of phenylmethyl silicone oil using an analytical balance and let it stand in a room temperature and constant temperature environment for 3 min to eliminate surface air bubbles. Then, we put the copper powder, MEG and graphite into the electric thermostatic blast drying oven (80 °C for 30 min) in advance to remove the moisture in the powder. Next, we added the copper powder into the high-speed disperser (1500 r/min) to perform mechanical stirring for 10 min to make it evenly dispersed. In addition, when preparing copper powder + MEG type EJC, MEG should be added into base oil in advance and stirred in the high-speed disperser (2200 r/min), followed by 30 min ultrasonic dispersion to prevent MEG agglomeration.

(3) Initial mixing: The sample mixing was accompanied by mechanical stirring throughout, and the filler equipped in advance was added to the base solution at a uniform rate. After the filler was mixed with phenylmethyl silicone oil, half of the thickening agent was added, and the solution became semisolid at this time. The mixture was stirred continuously until it softened to a paste and was left to stand for 3 min.

(4) Mixing and stirring: We put the preliminary mixed sample into the mixer (set three procedures: the first one, regular high-speed dispersion under atmospheric pressure for 80 s, rotational speed 1200 r/min, draw off the large air bubbles in the sample; the second procedure, vacuum high-speed dispersion for 120 s, rotational speed 1800 r/min, vacuum the air bubbles in the sample thoroughly; the third, release atmospheric pressure high-speed dispersion for 60 s, rotational speed 1500 r/min; the ratio of revolution to rotation for all three procedures is 1:0.5), during which the remaining thickening machine was added in two stages, waiting for the vacuum stirring to be complete, and left to cool naturally to room temperature to make the finished product of EJC.

### 2.4. Characterization

Characterization of the surface morphology of MEG using field emission scanning electron microscopy (SU8020, Hitachi, Tokyo, Japan), atomic force microscopy (Dimension Icon, Bruker, Germany), Raman scattering spectroscopy (Renishaw in Via, Renishaw, UK), and infrared spectroscopy (Nicolet IS10, Nicolet, Madison, WI, USA).

The automatic Tapered Entry Tester (WM-221A, Wanmu Instrument Co., Ltd., Guangdong, China) was used to measure the dropping point of EJC. The EJC was loaded into the cone tester at 25 °C; the 1/4 cone and cone rod combination piece, which was released from the cone penetration meter, made the cone fall for 5 s and measured the penetration depth. A total of three measurements were made on the samples, and the average value was taken.

A grease-dropping point tester (SYP4110-I, Shenkai Petroleum Instruments Co., Ltd., Shanghai, China) was used to determine the dropping point of MEG EJC. The sample of electric EJC was loaded into the grease cup of the dropping point tester, during which the equipment was energized and kept warming up, and the temperature of the sample when the grease dropped the first drop and reached the test tube during the test was observed and recorded, which was the measured dropping point.

The volumetric resistivity of the sample was determined by using a volumetric surface resistance tester (GEST-121, Guangshi Jingdian Instruments & Equipment Co., Ltd., Beijing, China), firstly cleaning the surface of the electrode plate with alcohol and then further cleaning the plate with deionized water after drying. The electrode plate was dried naturally and then filled with power compound until the electrode was full, and the excess power compound sample was scraped off. We added 500 V DC voltage to the test electrode plate, tested the resistance value of the test sample by measuring the leakage current perpendicular to the sample or along the surface of the sample and calculated the volume resistivity of the sample.

The friction coefficient of EJC was measured by using a microcomputer-controlled four-ball friction tester (MRS-10A, Vipin Testing Machine Co., Ltd., Jinan, China). Before the test, the steel balls were ultrasonically cleaned in petroleum ether for about 10 min. in the form of sliding friction, and the average friction coefficient and the diameter of the abrasion spots were detected. At the end of the test, the steel ball was removed and cleaned in the ultrasonic cleaner with petroleum ether for 5 min. The average grinding spot diameter of the steel ball was measured by a using digital metallographic microscope, and the surface morphology of the steel ball grinding marks was observed.

### 2.5. Application

Practical application of EJC in power systems: The surface of the copper–copper power joint busbar was polished and coated with EJC, and a tightening torque of 10 N was applied to the bolt between the overlap of the two plates. The cold contact resistance measured using a circuit resistance tester was less than 12 μΩ, which has good serviceability.

## 3. Results and Discussion

### 3.1. Morphology and Structure of MEG

**Figure 2:**
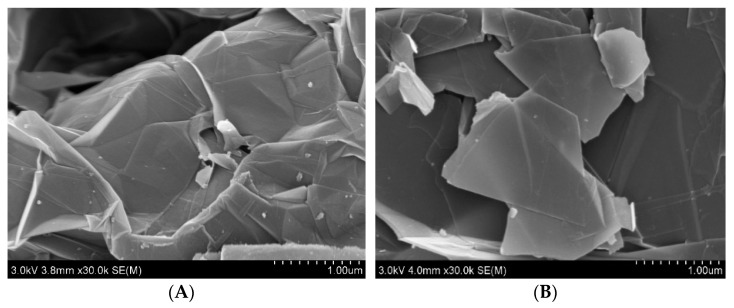
Expanded graphite and MEG 30,000× magnification. (**A**) Expanded graphite; (**B**) MEG.

Expanded graphite and MEG samples were observed using a field emission scanning electron microscope with the detection voltage set at 3.0 kV and the distance between the sample and the probe at 4 mm. The images of expanded graphite and MEG under field emission scanning electron microscopy with a magnification of 30,000 times are shown in Figure 2.

As shown in Figure 2a, the expanded graphite interlayer is closely aligned with darker color, but lighter separation areas appear at the edge locations. This is mainly due to the decrease of van der Waals forces between graphite flake layers due to the intercalation treatment, the decrease of crystallinity and the increase of layer spacing, which make the graphite more easily separated between layers during mechanical peeling. After the expanded graphite was homogenized under high pressure, the van der Waals forces between the layers were disrupted, and the flake MEG was separated and dispersed into independent flake structures under the action of ultrasonic waves, as shown in Figure 2B. The mechanically exfoliated MEG shows a highly translucent morphology with a high surface smoothness. The field emission scanning electron microscope images show that the MEG has high quality, thin thickness, large lateral dimensions and few in-plane defects.

**Figure 3:**
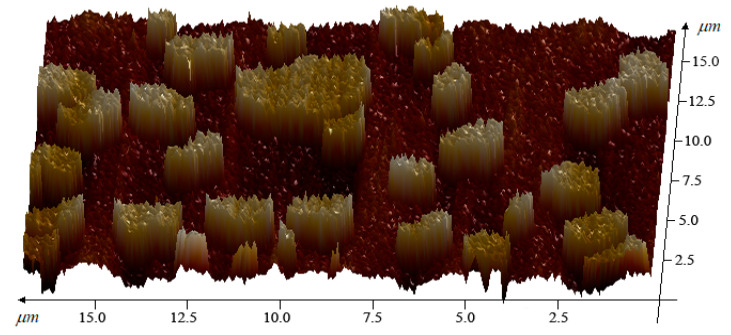
Expanded graphite and MEG 30,000× magnification.

To further quantify the thickness of mechanically exfoliated MEG samples and their lateral dimensions, the dispersed MEG flakes were observed using atomic force microscopy, and the resulting three-dimensional atomic force microscopy images of MEG are shown in Figure 3.

MEG is dispersed in small flakes with single lateral size between 0.5–5 μm and relatively uniform thickness; this large size flake structure is conducive to assisting metal fillers to form conductive pathways.

**Figure 4:**
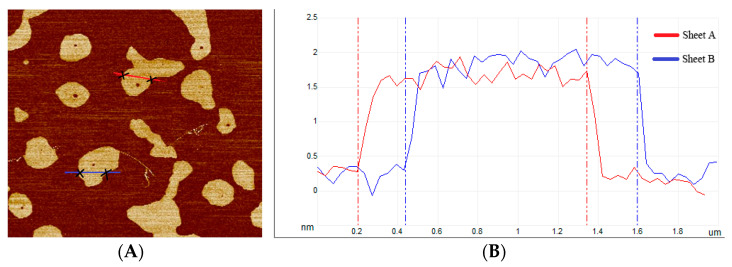
MEG sample cross-sectional scan waveform. (**A**) Cross-sectional selection; (**B**) Cross-sectional waveform.

Two MEG sheet sections were used as samples to measure the MEG thickness by atomic force microscopy; the test results are shown in Figure 4.

Figure 4A shows the microscopic images of the selected MEG samples, and Figure 4B shows the waveforms of the cross-sectional dimensions of the samples. The thicknesses of MEG in the sample sites are all around 2 nm, and it can be judged that the number of MEG layers in the sample sites is roughly 5 layers, which belongs to few-layer MEG. The thinner thickness is conducive to giving full play to the high carrier mobility of MEG, as well as reducing the potential barrier and enhancing the tunneling effect between MEG and copper, which effectively improves the electrical conductivity of the power composite grease. The comprehensive results show that the MEG is in good condition.

**Figure 5:**
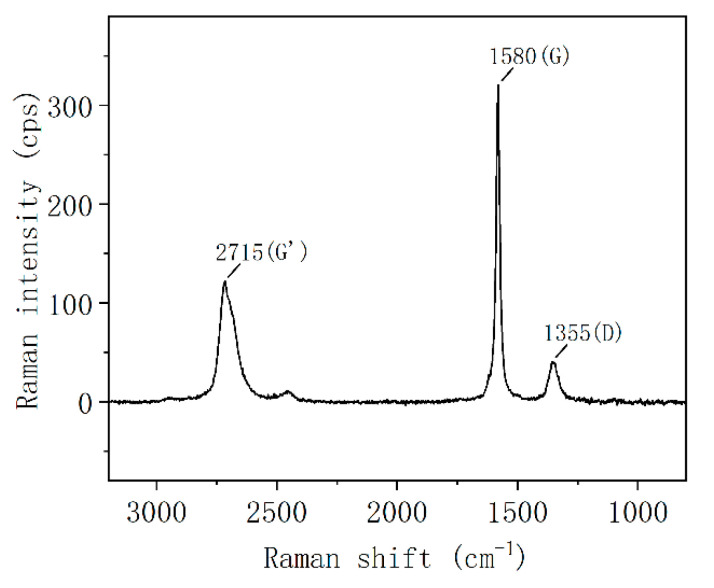
The Raman spectra of mechanically exfoliated MEG samples.

Defects are also an important indicator of MEG properties. To further investigate the condition of defects within the face of mechanically exfoliated MEG, MEG was analyzed using Raman scattering spectroscopy. The Raman spectra of mechanically exfoliated MEG samples are shown in Figure 5. There are three Raman bands at approximately 1355 cm^−1^ (D), 1580 cm^−1^ (G) and 2710 cm^−1^ (G’). The intensity of the G peak is higher than that of the G’ peak, while the G and G’ peaks are red- and blue-shifted, respectively. This indicates that this MEG sample has a multilayer structure, and the number of layers is greater than four [16]. The number of MEG layers can be estimated to be 6 by measuring the G-peak intensity [17], which is in good agreement with the AFM measurements. The D-peak in the spectrum characterizes the structural defects in MEG, and the intensity ratio *n_D_* of D-peak to G-peak characterizes the defect density in MEG, with the relation shown in Equation (1) [18]:

(1) $$n_{D}({\mathrm{cm}}^{-2})=(7.3\pm 2.2)*10^{9}E_{L}^{4}\left(\frac{ID}{IG}\right)$$

where ID and IG are the intensities of the D-peak and G-peak of the Raman spectrum, and $E_{L}$ is the laser energy. From Equation (1), the D-peak intensity is proportional to the defect density at a determined laser energy. The lower D-peak intensity can be seen from the spectrum, which indicates that the mechanically exfoliated MEG has fewer in-plane defects and higher surface integrity.

**Figure 6:**
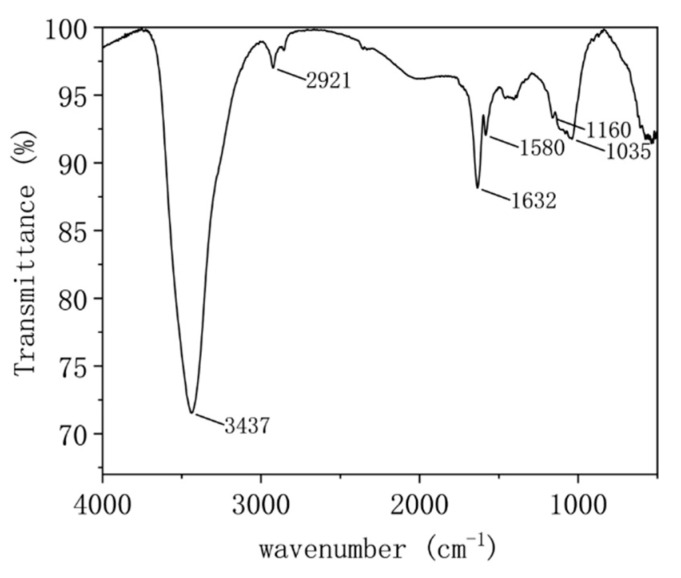
Infrared spectrogram of MEG.

Infrared spectroscopy characterization was performed to assess the surface functionalization of the MEG (Figure 6). A distinct C=C bond vibration characteristic peak appears near 1580 cm^−1^, which characterizes the MEG skeleton structure. In addition, the -CH2 methylene symmetric stretching vibration peak near 2855 cm^−1^ appears in the Figure 6, which is considered to be caused by the unsaturated carbon atoms at the edge of MEG saturated by hydrogen, and the alcohol-based vibration peak is found at 1160 cm^−1^–1040 cm^−1^, which is considered to be caused by the sp3 hybridization defect at the edge of MEG and saturated by hydroxyl group. The –OH hydroxyl stretching vibration peak near 3437 cm^−1^ and the –OH hydroxyl bending vibration peak near 1632 cm^−1^ are mainly due to the effect of water molecules in air and water used in the preparation of MEG.

From the above characterization, we conclude that the prepared MEG has a large lateral size and few-layer structure, with fewer defects in the face layer, which can effectively improve the electrical conductivity in lipid solvents. However, due to the intercalation operation of expanded graphite, the MEG defects introduced a small amount of hydroxyl and methylene groups, which may improve the dispersion performance of MEG in solvents.

### 3.2. Characterization of Physicochemical Properties of MEG Power Composite Grease

#### 3.2.1. Penetration

**Figure 7:**
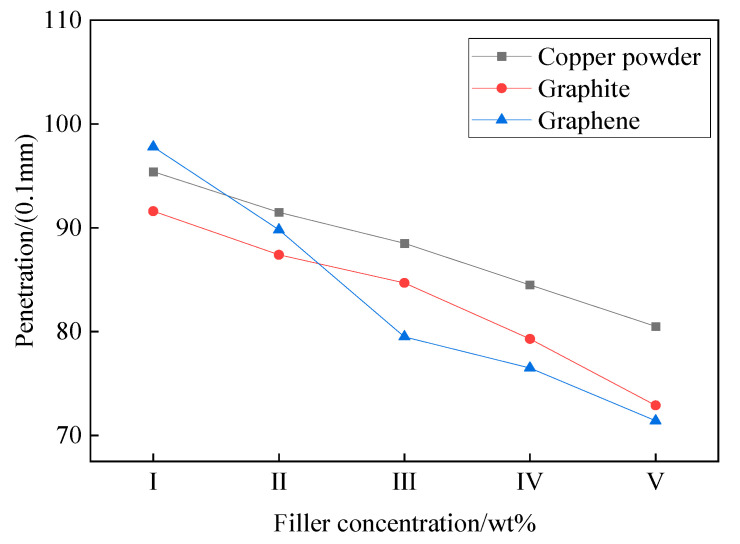
The relationship between penetration and filler content of MEG power complex grease.

Penetration is measured by detecting the depth of the tip cone into the test object under gravity and is primarily used to characterize the hardness of the EJC. According to the requirements of compound grease use in actual engineering, the nonworking penetration of compound grease (25 °C, 1/4 cone and cone rod assembly, 5 s drop, vertebra drop depth) is usually between 70 and 90. After testing all configurations of paste, the relationship between penetration and filler content of MEG power complex grease is shown in Figure 7.

From Figure 7, it can be found that the filler content has a great influence on the cone penetration of EJC, and the cone penetration of all three groups of compound grease samples showed a decreasing trend with the increase of filler content. Among them, the cone penetration of MEG power grease decreased the most significantly with 26.4. The cone penetration of graphite power grease and copper powder power grease decreased by 18.7 and 14.9, respectively. The reason is that the cone penetration is influenced by the particle size and specific surface area of the filler: the larger the specific surface area and the higher the hardness of the material, the greater the reduction of the cone it becomes. The specific surface area of the conductive filler used in the experiment is copper powder < graphite < MEG, which is consistent with the experimental data. The cone penetration mainly reflects the softness and hardness of EJC, and the range of cone penetration in engineering application is between 70 and 90. If the EJC is too soft (cone penetration >90), the viscosity of the grease is low, and it is easy to be lost under high temperature. If the EJC is too hard (the cone penetration is less than 70), it is easy to blister and dry up during the actual application process, resulting in incomplete contact with the electrical connection position. From the experimental data, it can be seen that the penetration is within a reasonable range when the MEG content is 2 wt%–4 wt%, that the MEG has a high penetration adjustment rate and adjustment range and that a small change of MEG content can effectively adjust the penetration, which is conducive to help balance the properties of EJC.

#### 3.2.2. Drip Point

**Figure 8:**
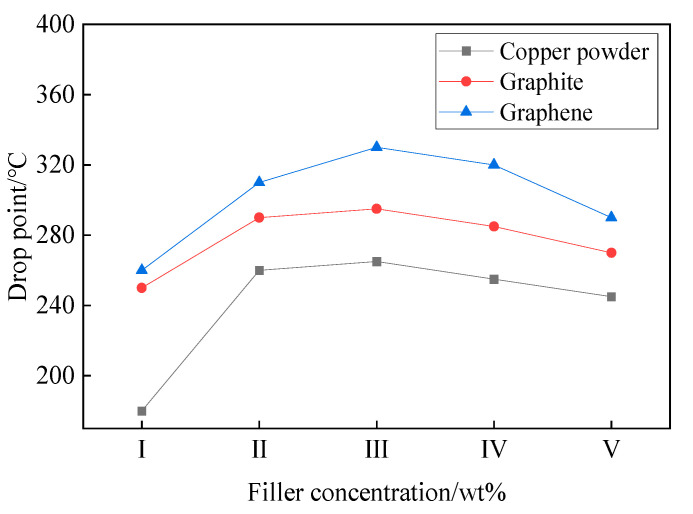
Different filler concentration–dropping point relationship.

The higher the dropping point is, the more stable the performance of the grease in the high-temperature environment. When the grease works at a temperature higher than its own limit, it will cause the separation of oil and grease and lead to the failure of the grease. The factors that affect the dropping point size are mainly the performance of conductive filler, the performance of base oil, the performance of thickener and the control of preparation process. The experimental measurement of different filler concentration–dropping point relationship is shown in the Figure 8.

As shown in Figure 8, with the increase of filler content, the dropping points of all three groups of electric power composite grease showed a trend of first increasing and stabilizing and then gradually decreasing. The trends are due to the fact that when the filler concentration is low, the viscosity of the compound grease is low and easy to flow, and when the content of conductive filler is continuously increased, the grease starts to harden, which destroys the internal colloidal stability of the compound grease. Therefore, at a higher concentration of conductive filler, the dropping point appears to decrease to some extent. At the same filler ratio, MEG-added compound grease has the highest dropping point compared to pure copper fraction and graphite-added compound grease, and the dropping point reaches the maximum value of 330 degrees at 3 wt% of MEG filler. This indicates that MEG addition can effectively improve the thermal stability of the composite grease.

#### 3.2.3. Volumetric Resistivity

**Figure 9:**
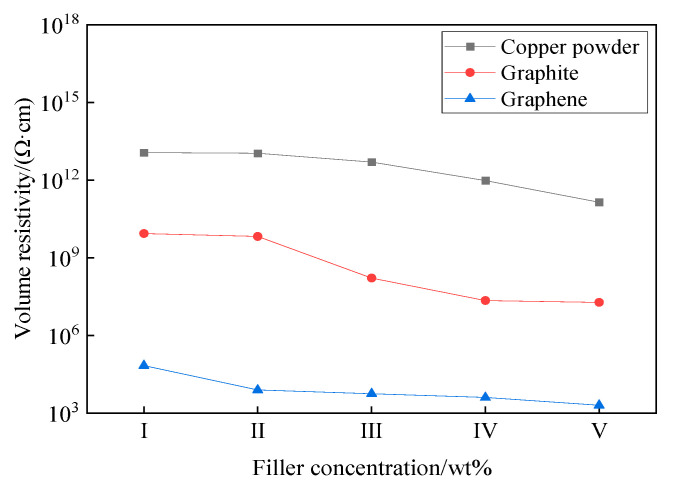
Volume resistivity–filler concentration relationship.

When the EJC works normally, there are mainly two types of conductive methods: one is that some of the conductive particles will be in direct contact, forming a continuous chain-like conductive path, and the other is that some of the conductive particles are very close to each other, forming a tunneling current under the action of “tunneling effect” [19]. Volume resistivity is the impedance of a material to current per unit volume. It can effectively characterize the ability of conductive fillers to build conductive pathways and form tunneling currents. The volume resistivity–filler concentration relationship is shown in Figure 9.

It can be concluded from the data that the volume resistivity of MEG power complex grease is eight orders of magnitude and four orders of magnitude lower compared with copper powder type power complex grease and graphite type power complex grease, respectively. The specific surface area of MEG with large lateral size and nanometer thickness is much larger than the other two conductive fillers, which makes more MEG aggregates of the same mass; the conductive particle spacing will be smaller; and it is easier to form conductive pathways. The sheet-like structure of MEG can effectively reduce the tunneling barriers [20], which in turn reduces the bulk resistivity by lowering the tunneling resistance. The characteristics of volume resistivity can reflect to some extent that MEG power complex grease has stronger electrical conductivity compared with copper powder and graphite-added power complex grease.

#### 3.2.4. Frictional Properties

The friction and wear performances of EJC are both very important index to judge the quality of EJC. The size of friction coefficient of electric EJC determines the quality of its wear reduction and fatigue resistance performance in actual working condition, and the size of wear volume determines the superiority of its antiwear performance.

**Figure 10:**
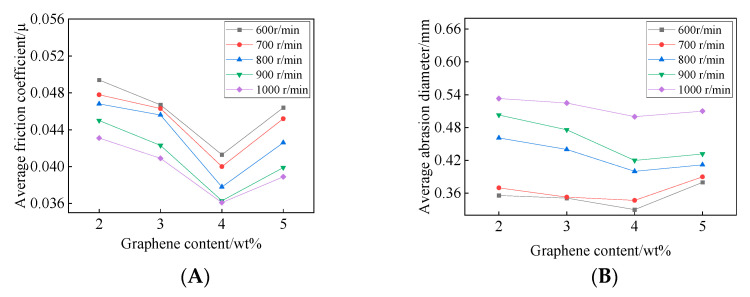
The relationship between the frictional properties of the EJC and the MEG. (**A**) Average friction coefficient; (**B**) Average abrasion diameter.

The relationship between the frictional properties of the EJC and the MEG content at different rotational speeds under a fixed load of 392 N is shown in Figure 10.

Figure 10a shows the relationship between the average friction coefficient and MEG content. With the increase of MEG content, the friction coefficients of all electric power complex greases decreased first and then increased. When the MEG content was 4 wt%, the friction coefficient was the smallest, and the electric power complex grease had the best friction reduction performance. The wear diameters of the three steel balls after the four-ball friction test were observed with a high-definition CCD (charge coupled device camera) measuring instrument. As shown in Figure 10b, with the increase of MEG content, the average wear diameter of the steel balls under the five electric power composite greases decreased first and then increased, and the average wear diameter was the smallest when the MEG content was 4 wt%.

The effect of speed on friction coefficient and wear spot size is due to the fact that at very slow speed, there is dry friction between the shaft and the shaft tile. As the speed increases, the friction mode gradually changes to boundary friction (due to the adsorption of lubricant and metal surface, a very thin boundary oil film is formed on the metal surface) and fluid friction (the two contact surfaces are completely separated by a continuous film of lubricant), both of which have small coefficient of friction. As the speed continues to increase, the dynamic pressure oil film is formed, making the lowest coefficient of friction.

## 4. Conclusions

The mechanically exfoliated graphene was prepared by high-vacuum shear grinding and high-pressure homogenization method using expanded graphite as the raw material. The prepared samples were characterized by field emission scanning electron microscopy, atomic force microscopy, Raman spectroscopy and infrared spectroscopy. The average number of layers of the prepared mechanically exfoliated graphene was found to be 5–6, and the transverse dimensions of the single flakes were 0.5μm–5μm, with smooth surfaces and less in-plane defects.

The electrical joint compound was prepared with phenylmethyl silicone oil as the base oil and copper and graphite and graphene as the conductive fillers. The cone penetration, dropping point, volume resistivity and friction properties of the electrical joint compound were characterized. It was found that the cone penetration of the power complex grease decreased with the increase of the filler ratio, and the cone penetration decrease was more obvious due to the change of graphene ratio. The dropping point of the electrical joint compound increased first and then decreased with the increase of filler, among which the electrical joint compound filled with mechanically exfoliated graphene had higher dropping point, and the highest dropping point was at 3 wt% of graphene ratio. The volume resistivity of the graphene power loading grease also decreased significantly compared to the other controls. In addition, the graphene filler greatly optimized the frictional properties of the electrical joint compound, possessing the smallest average friction coefficient and wear spot size at a graphene concentration of 4 wt%.

In summary, it was found that the graphene prepared by the mechanical peeling method causes a significant improvement of the electrical joint compound penetration, drop point, volume resistivity and friction properties, which are all beneficial to enhance the contact performance of electrical joints and guarantee the stable and efficient transmission of electric power.
